# Sevoflurane participates in the protection of rat renal ischemia‐reperfusion injury by down‐regulating the expression of TRPM7

**DOI:** 10.1002/iid3.753

**Published:** 2022-12-31

**Authors:** Xudong Xu, Rongrong Deng, Lu Zou, Xiaoyan Pan, Zhifeng Sheng, Da Xu, Tingting Gan

**Affiliations:** ^1^ Department of Anesthesiology Changzhou Hospital of Traditional Chinese Medicine Changzhou Jiangsu China

**Keywords:** inflammatory factor, oxidative stress, renal ischemia‐reperfusion injury, sevoflurane, TRPM7

## Abstract

**Introduction:**

To investigate the protective effect of sevoflurane preconditioning on renal ischemia‐reperfusion injury (renalischemiareperfusionmodel, RIRI) and its related mechanism.

**Methods:**

Eighty healthy adult male SD rats were randomly divided into control group (Sham group), model group (RIRI group), sevoflurane pretreatment group (Sev group) and TRPM7 inhibitor combined with sevoflurane pretreatment group (T + Sev group), 20 animals in each group. Hematoxylin‐eosin (HE) staining was used to observe the pathological changes of renal tissue, and the levels of creatinine and urea nitrogen in each group were detected. Deoxyribonucleic acid terminal transferase‐mediated dUTP nick end labeling (TUNEL) assay was used to detect renal cell apoptosis, and Western blottingwas used to detect the expression of apoptotic proteins cleaved‐caspase‐3, bax, Bcl‐2, and TRPM7 in renal tissue; Detection of oxidative stress‐related index levels in renal tissue and levels of inflammatory factors in renal tissue and serum.

**Results:**

Compared with the Sham group, the renal tissue pathological damage was aggravated, the levels of creatinine and blood urea nitrogen were increased, and the apoptosis was increased in the RIR group and the Sev group. Death, malondialdehyde (MDA) levels and inflammatory factors were increased, and superoxide dismutase (SOD) levels were decreased (all *p* < .05); The scores, apoptosis rate, MDA level, and relative expression of inflammatory factor levels were decreased, and SOD levels were increased (all *p* < .05). Compared with the Sev group, the renal tissue pathological damage in the T + Sev group was aggravated, creatinine, blood urea nitrogen levels increased, apoptosis increased, apoptosis‐related proteins cleaved‐caspase‐3, bax, Bcl‐2 showed increased apoptosis, malondialdehyde (MDA) levels, inflammatory factor levels increased, ultrahigh The levels of oxide dismutase (SOD) were decreased (all *p* < .05).

**Conclusions:**

Therefore, we believe that sevoflurane is involved in the protection of rat renal ischemia‐reperfusion injury by downregulating the expression of TRPM7.

## Graphical abstract

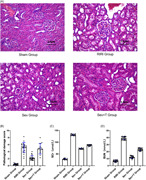

## 1 INTRODUCTION

Renal ischemia‐reperfusion injury (RIRI) refers to the renal ischemia caused by the aggravation of damage and deterioration of renal function (i.e., ischemia and hypoxia of renal tissue cells, renal tubular cell damage) after complete or partial restoration of blood perfusion in the ischemic kidney damage.^1^, ^2^ Renal ischemia‐reperfusion injury is common in patients with cardiovascular surgery, trauma, shock and renal transplantation, and is the most common cause of acute kidney injury in clinical practice. At present, there is no effective treatment strategy to cause renal ischemia‐reperfusion injury, which mainly includes energy metabolism disorders, calcium overload, mitochondrial damage, oxidative stress, inflammation, apoptosis, and necrosis.^3^, ^4^, ^5^ Therefore, in‐depth understandings of the mechanism of RIRI and improvement or reversal of its damage are important issues that need to be solved clinically.

As an inhaled anesthetic commonly used in general anesthesia, sevoflurane has the advantages of rapid induction of anesthesia, less irritation to the patient's respiratory tract, and rapid and complete recovery.^6^, ^7^, ^8^ The research results of some scholars have shown that sevoflurane has a certain protective effect on pulmonary RIRI, but the specific molecular mechanism is not fully understood.^9^ Studies by some scholars have shown that sevoflurane has a positive protective effect on CIRI by inhibiting apoptosis receptors and peroxisome proliferator receptors and reducing the levels of apoptosis and oxidative stress.^10^, ^11^, ^12^

In recent years, it has been found that transient receptor potential channel M7 (TRFM7) is an important nonselective calcium and magnesium ion channel expressed on proximal renal tubular epithelial cells. It also has kinase activity and can mediate intracellular and extracellular signal transduction. Various biopathophysiological processes of cells.^13^, ^14^ TRPM7 is abundantly expressed in most organs, especially in the kidney. As an ion channel, TRPM7 can nonselectively allow the passage of divalent cations such as calcium and magnesium, thereby depolarizing excitatory cells and leading to intracellular calcium overload; as a kinase, TRPM7 can phosphorylate itself or activate its substrates Annexin AI (AnnexinI1, myosin II and m‐calDain^15^, ^16^, ^17^). In the process of brain and myocardial injury, the increased expression of TRPM7 leads to the apoptosis of neurons and cardiomyocytes, while inhibition of TRPM7 can alleviate the stroke and myocardial injury.^18^, ^19^ However, the expression of TRPM7 in renal ischemia‐reperfusion injury has not been reported in detail. Therefore, this study established a rat model to explore the role of TRPM7 in rat renal ischemia‐reperfusion injury.

## 2 MATERIALS AND METHODS

### 2.1 Experimental animals and groups

Eighty healthy male adult SD rats, 6–8‐week‐old, weighing 220–320 g, were provided by Experimental Animal Center of Jiangsu University. All experimental animals were kept in a suitable ventilated environment (relative humidity of 40%–50%, room temperature at 22°C–24°C, light: dark cycles of 12 h light and 12 h darkness), and all animals were free to drink tap water and feed. Feed was provided by Jiangsu Synergetic Pharmaceutical Bioengineering Co., Ltd. SD rats were randomly divided into control group (Sham group), model group (RIRI group), sevoflurane pretreatment group (Sev group), and TRPM7 inhibitor group (Sev + T group), 20 rats in each group. Rats in the Sham group were treated with sham surgery. The renal ischemia‐reperfusion model was established in the RIRI group. Rats in the Sev group were intervened with sevoflurane 1 h before the establishment of the pulmonary IRI model. TAK‐242 (10 mg/kg) was injected into the tail vein of the rats in the T + Sev group, followed by sevoflurane intervention 1 min later, and a renal RIRI model was established 1 h later. This study was approved by the Ethics Committee of Changzhou Hospital of Traditional Chinese Medicine (approval number: XJTU2AF201‐08).

### 2.2 Main reagents and instruments

Sevoflurane was purchased from Wuhan Aimeijie Technology Co., Ltd.; 10% chloral hydrate was purchased from Shanghai Sinopharm Chemical Reagent Co., Ltd. Deoxyribonucleic acid terminal transferase‐mediated dUTP nick‐end labeling (terminaldeoxynucleotidyltransferase‐mediated dUTPnick‐end labeling, TUNEL) staining The kit and enzyme‐linked immunosorbent assay (ELISA) kit were purchased from Beijing Soleibao Technology Co., Ltd.; cleaved‐caspase‐3, bax, Bcl‐2, TRPM7 antibody, β‐actin antibody, and secondary antibody were all Purchased from Abcam; fully automatic biochemical analyzer was purchased from Berkam.

### 2.3 Animal modeling and specimen collection

Experimental rats were injected intraperitoneally with 10% chloral hydrate 0.3 ml/100 g. After the depth of anesthesia reached the surgical requirements, the rats were dehaired and placed under a warm light on the operating table. The skin, rectus abdominis and peritoneum were incised layer by layer along the midline of the upper abdomen, the left and right kidneys were exposed, the renal pedicle was freed, the renal pedicle was clamped to block the renal blood flow for 45 min, and then released to allow the blood flow to recanalize, and the color change of the kidney was observed. The kidneys changed from dark purple to red within 5 min, indicating that the ischemia‐reperfusion injury model was successfully established.^20^ After naturally awakening, the kidneys were placed in a relatively clean and constant temperature metabolic cage, received urine for 24 h, fed with standard chow, and freely drank water; after 5 min, the kidneys were if it does not turn into normal red, the model establishment fails and it is excluded from the experiment. Sevoflurane intervention: The rats were placed in a self‐made airtight experimental chamber, the concentration of sevoflurane was adjusted to 2.1%, the concentration of oxygen was 21%, and normal ventilation was resumed for 30 min after pretreatment for 30 min.

## 3 EXPERIMENTAL METHODS

### 3.1 Pathological examination

Lung tissues fixed in 4% paraformaldehyde solution for 24 h were taken to make paraffin sections and stained with hematoxylin‐eosin (HE). After drying, the sections were dewaxed with xylene, washed with ethanol gradient, stained with hematoxylin for 5 min, differentiated with hydrochloric acid and ethanol, stained with eosin for 1 min, rinsed with tap water, dehydrated with ethanol gradient, transparent with xylene, and mounted with neutral resin. The pathological changes of renal tissue were observed by light microscope.

### 3.2 TUNEL assay to detect apoptosis in renal tissue

TUNEL apoptosis detection kit was used to detect the apoptosis of renal tissue cells and calculate the apoptosis rate. The kidney tissue paraffin sections were deparaffinized and rehydrated, digested with proteinase K, incubated in the dark after adding TUNEL reaction buffer, 4ʹ,6‐diamidino‐2‐phenylindole (4ʹ,6‐diamidino‐2‐ Nuclei were counterstained with phenylindole, DAPI) and mounted with antifluorescence quencher. The apoptosis of each group was observed under a fluorescence microscope, and the positive cells showed red fluorescence. Five slices were selected in each group, and five fields of view (×200) were randomly selected for each slice.

### 3.3 Detection of the expression of related proteins by western blotting

Partially cryopreserved lung tissue was taken, ground in liquid nitrogen, and fully lysed by adding RIPA protein lysing solution to extract total protein. The protein concentration was determined by BCA method. The protein samples were electrophoresed through a polyacrylamide gel, and then blocked with 5% skim milk at room temperature for 1.5 h after transfer. Primary antibodies [cleaved‐caspase‐3 antibody (1:1000), bax antibody (1:1000), Bcl‐2 antibody (1:1000), TRPM7 antibody (1500), β‐actin antibody (12000)] were incubated at 4°C overnight, and the secondary antibody (11000) was incubated at room temperature for 1 h, and ECL substrate was added dropwise for luminescence and color development. The grayscale of the bands was detected by a gel image analysis system, and the relative expression level of the target protein was calculated.

### 3.4 Detection of oxidative stress‐related indicators in kidney tissue

Part of the cryopreserved lung tissue was taken, homogenized by adding nine times normal saline, and the supernatant was collected after centrifugation for detection. The level of malondialdehyde (MDA) was detected by thiobarbituric acid colorimetric method, and the level of total superoxide dismutase (SOD) was determined by WST‐8 method.

### 3.5 Detection of inflammatory factors in kidney tissue and serum

The levels of interleukin (IL)‐6 and tumor necrosis factor (TNF)‐α in serum were detected by ELISA. The level of myeloperoxidase (MPO) in lung tissue was determined by dianisidine colorimetric method.

### 3.6 Statistical methods

Statistical analysis was performed using SPSS 19.0 software. The measurement data conforming to the normal distribution were expressed as mean ± standard deviation, the comparison between multiple groups was performed by one‐way analysis of variance, and the comparison between groups was performed by LSD‐t test. *p* < .05 means the difference is statistically significant.

## 4 RESULTS

### 4.1 Effect of sevoflurane on pathological injury of renal tissue

**Figure 1:**
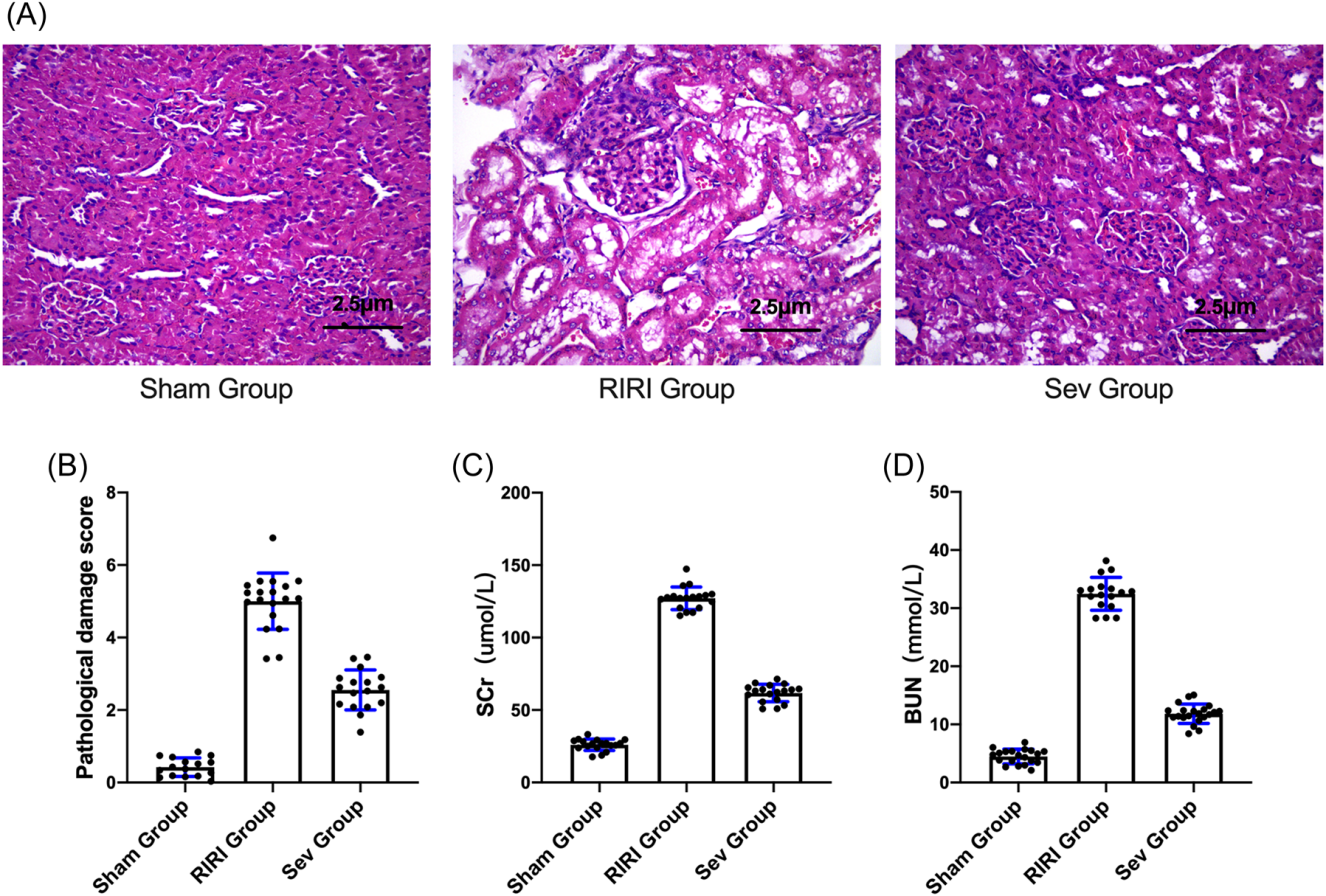
The effect of sevoflurane on pathological injury of renal tissue. (A) HE staining for the morphological and structural changes of kidney tissue. (B) Rat kidney pathological damage score. (C) Changes in serum creatinine of rats in each group. (D) Changes in serum urea nitrogen of rats in each group. BUN, blood urea nitrogen; RIRI group, renal ischemia‐reperfusion injury group; Scr, Creatinine; Sev group, sevoflurane treatment group; Sham group, sham operation group; *p < .05 compared with Sham group. ^#^ *p* < .05 compared with RIRI group.

The results of HE staining showed that the morphological structure of the kidney in the Sham group was normal. RIR caused marked renal histomorphological changes, including tubular dilation, flattening or disappearance of the brush border, degeneration and necrosis of some tubular epithelial cells, exposure of the basement membrane and infiltration of mononuclear cells, and the necrotic and exfoliated cellular debris fell into the lumen (tube type) and so forth. The Sev group significantly improved RIR‐induced histological changes (Figure 1A). Compared with the Sham group, the renal pathological damage scores of the RIRI group and the Sev group were increased (*p* < .05). Compared with the RIRI group, the renal pathological damage score of the rats in the Sev group was decreased (*p* < .05) (Figure 1B).

### 4.2 Changes of renal function indexes of rats in each group

Compared with the Sham group, the serum creatinine of the RIRI group and the Sev group increased (*p* < .05). Compared with the RIRI group, the serum creatinine of the Sev group was decreased (*p* < .05) (Figure 1C). Compared with the Sham group, the serum urea nitrogen in the RIRI group and the Sev group increased (*p* < .05). Compared with the RIRI group, the serum urea nitrogen in the Sev group was decreased (*p* < .05) (Figure 1D).

### 4.3 The effect of sevoflurane on apoptosis of renal tissue cells

**Figure 2:**
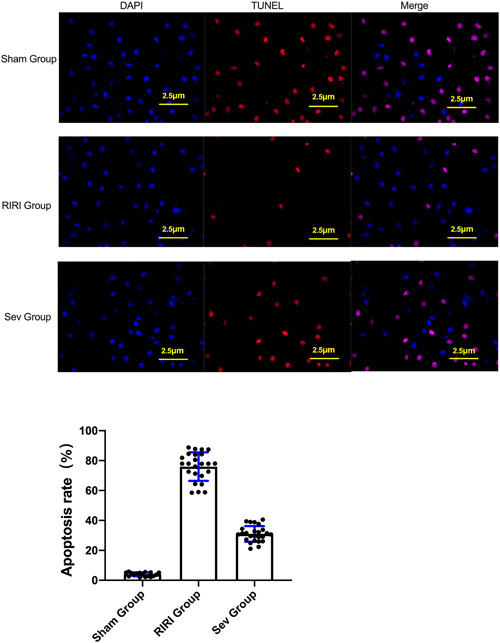
The effect of sevoflurane on apoptosis in renal tissue. (A) TUNEL apoptosis assay. (B) Comparisn of apoptosis rate of rats in each group. RIRI group, renal ischemia‐reperfusion injury group; Sev group, sevoflurane treatment group; Sham group, sham operation group; *p < .05 compared with Sham group. ^#^ *p* < .05 compared with RIRI group.

The TUNEL cell apoptosis test showed that compared with the Sham group, the apoptosis rate of the kidney tissue of the rats in the RIRI group and the Sev group was increased (*p* < .05) (Figure 2A). Compared with the RIRI group, the apoptosis rate of renal tissue in the Sev group was lower than that in the RIRI group (*p* < .05) (Figure 2B).

**Figure 3:**
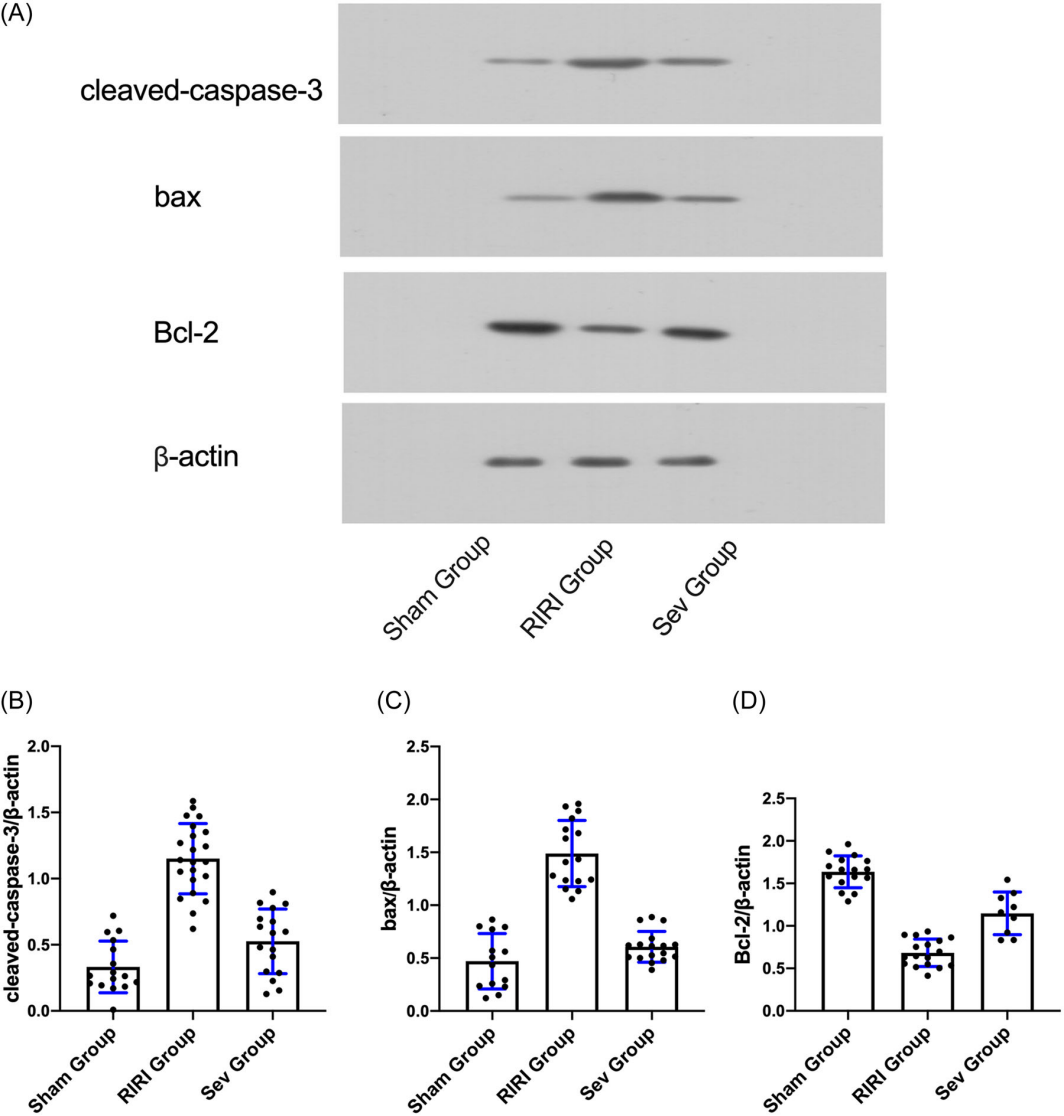
Apoptosis‐related protein expression. (A) Expression of apoptosis‐related protein cleaved‐caspase‐3; (B) Expression of apoptosis‐related protein bax. (C) Expression of apoptosis‐related protein Bcl‐2. RIRI group, renal ischemia‐reperfusion injury group; Sev group, sevoflurane treatment group; Sham group, sham operation group. *p < .05 compared with Sham group. ^#^ *p* < .05 compared with RIRI group.

WB results showed that compared with the Sham group, the expressions of apoptosis‐related proteins cleaved‐caspase‐3 and bax in the kidney tissue of the RIRI group and Sev group were increased (*p* < .05), and the expression of Bcl‐2 was decreased (*p* < .05). Compared with the RIRI group, the expressions of apoptosis‐related proteins cleaved‐caspase‐3 and bax in the kidney tissue of the Sev group were lower than those of the RIRI group (*p* < .05), and the expression of Bcl‐2 was higher than that of the RIRI group (*p* < .05). The apoptosis of kidney tissue of rats in RIRI group and Sev group increased, and sevoflurane could improve the apoptosis of renal cells caused by renal ischemia‐reperfusion (Figure 3D).

### 4.4 Expression of TRPM7 protein in rats in each group

**Figure 4:**
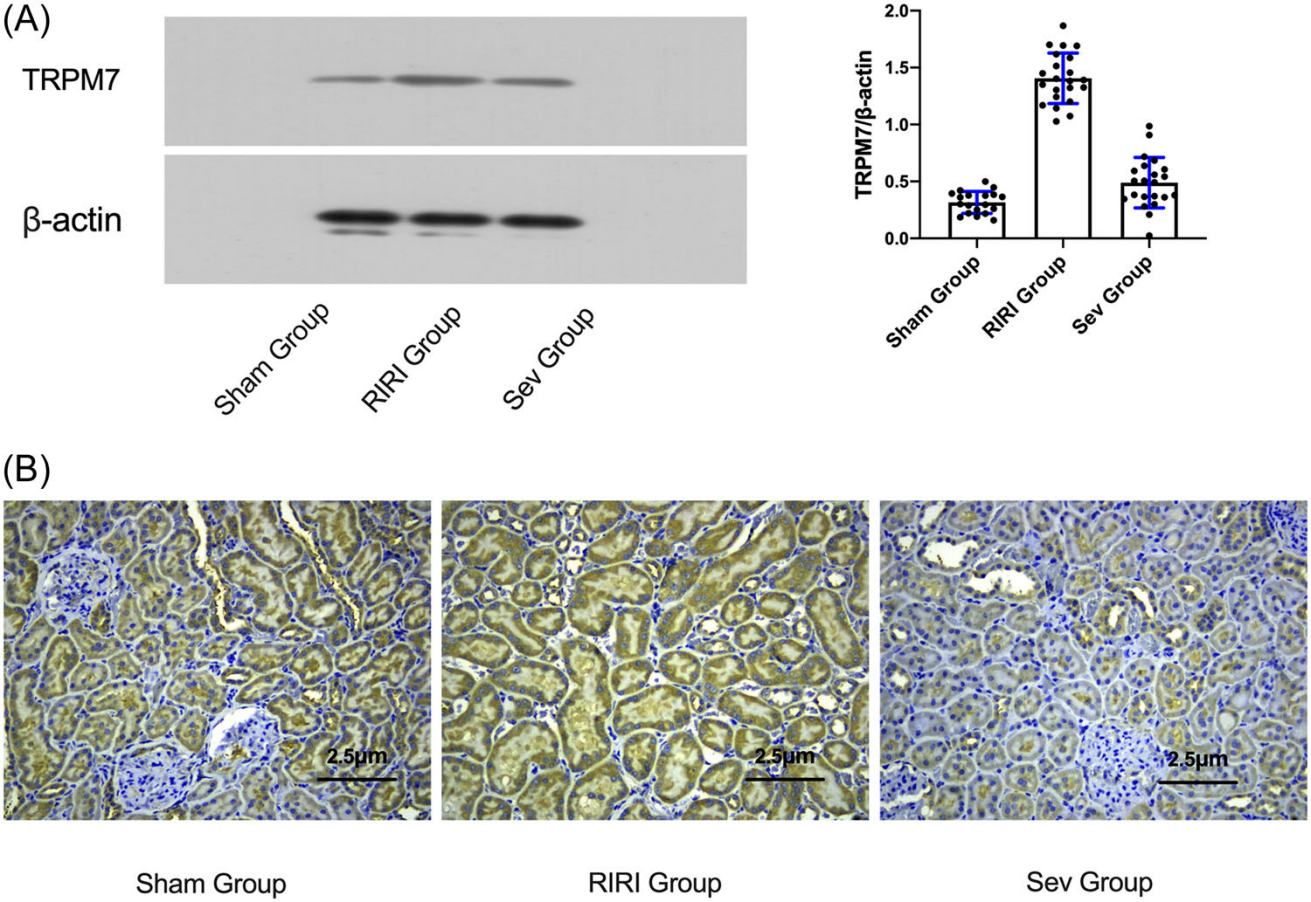
TRPM7 expression and localization. (A) The expression of TRPM7 protein in each group of rats; (B) The expression and localization of TRPM7 protein in rat kidney tissue after I/R were detected by immunohistochemical method. RIRI group, renal ischemia‐reperfusion injury group; Sev group, sevoflurane treatment group; Sham group, sham operation group. *p < .05 compared with Sham group. ^#^ *p* < .05 compared with RIRI group.

WB results showed that compared with the Sham group, the TRPM7 protein concentration in the kidney tissue of the RIRI group and the Sev group was increased (*p* < .05). Compared with the RIRI group, the cellular TRPM7 protein concentration in the renal tissue of the Sev group was decreased (*p* < .05) (Figure 4A).

### 4.5 Immunohistochemical method to detect the expression and localization of TRPM7 protein in rat kidney tissue after I/R

TRPM7 was mainly expressed on proximal tubular epithelial cells. Compared with the Sham group, the expression of TRPM7 on the renal tubular epithelial cells in the RIRI group and Sev group was significantly increased (*p* < .05). The expression of TRPM7 on renal tubular epithelial cells was decreased (*p* < .05) (Figure 4B).

### 4.6 The effect of sevoflurane on the pathological injury of renal tissue after regulating the expression of TRPM7 protein

**Figure 5:**
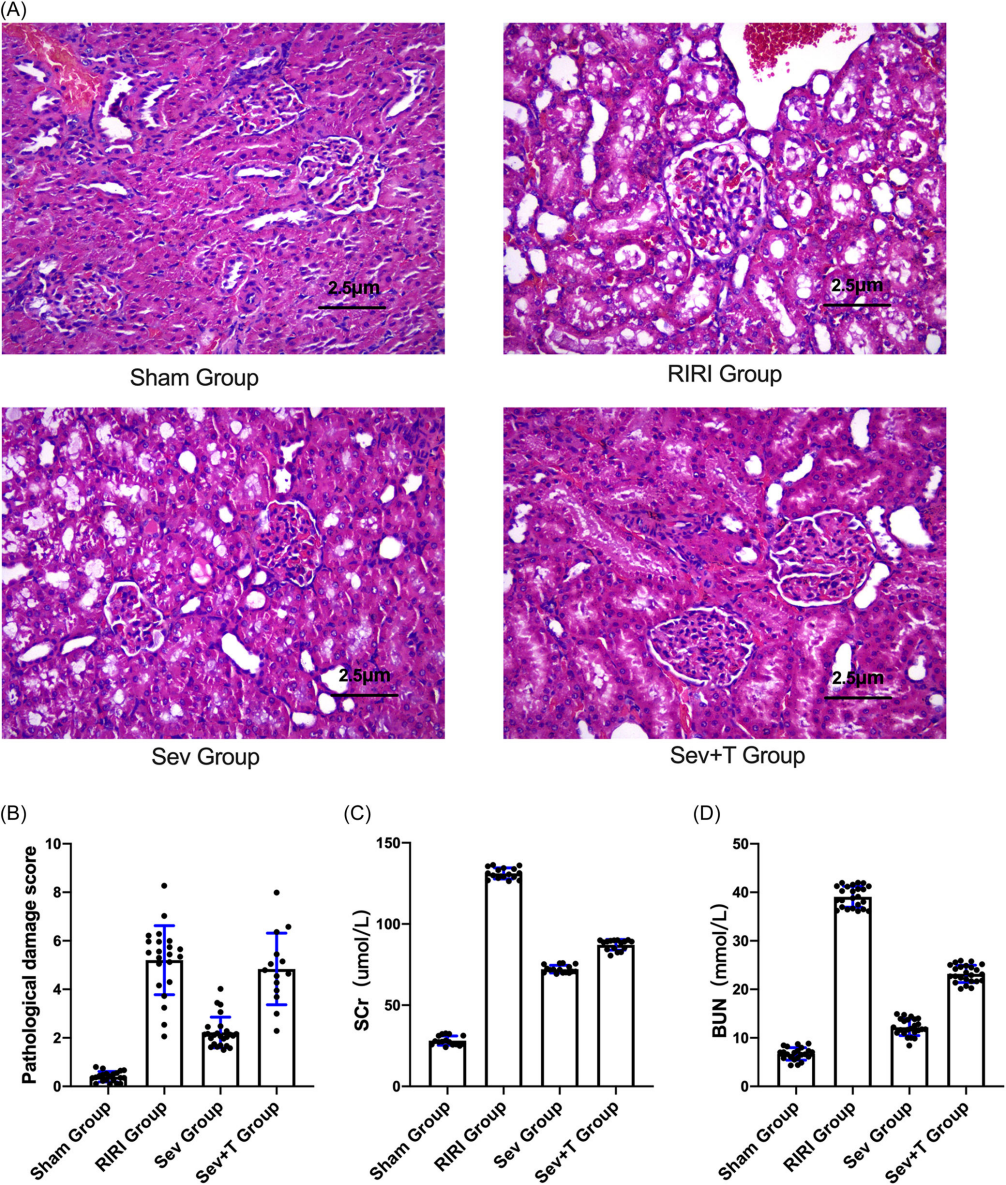
Effect of sevoflurane on pathological injury of renal tissue after regulating TRPM7 protein expression. (A) HE staining for the morphological and structural changes of kidney tissue. (B) Rat kidney pathological damage score. (C) Changes in serum creatinine of rats in each group. (D) Changes in serum urea nitrogen of rats in each group. BUN, blood urea nitrogen; RIRI group, renal ischemia‐reperfusion injury group; Scr, Creatinine; Sev group, sevoflurane treatment group; Sev + T group, sevoflurane treatment after inhibiting the expression of TRPM7 Group; Sham group, sham operation group. *p < .05 compared to the Sham group. ^#^ *p* < .05 compared with RIRI group. ^&^ *p* < .05 compared with Sev group.

The results of HE staining showed that the morphological structure of the kidney in the Sham group was normal. RIR caused marked renal histomorphological changes, including tubular dilation, flattening or disappearance of the brush border, degeneration and necrosis of some tubular epithelial cells, exposure of the basement membrane and infiltration of mononuclear cells, and the necrotic and exfoliated cellular debris fell into the lumen (tube type) and so forth. The Sev group significantly improved the histological changes caused by RIR, and after inhibiting TRPM7, the protective effect of sevoflurane on renal tissue was significantly reduced (Figure 5A,B).

### 4.7 Changes of renal function indexes of rats in each group after adjusting the expression of TRPM7 protein, creatinine, and urea nitrogen

Compared with the Sham group, the serum creatinine of the RIRI group, the Sev group and the Sev + T group increased (*p* < .05). Compared with the RIRI group, the serum creatinine of the Sev group was decreased (*p* < .05). After inhibiting TRPM7, the effect of sevoflurane on reducing the rat creatinine was significantly reduced (*p* < .05). (Figure 5C). Compared with the Sham group, the serum urea nitrogen in the RIRI group and the Sev group increased (*p* < .05). Compared with the RIRI group, the serum urea nitrogen in the Sev group was lower, and the effect of sevoflurane on reducing the urea nitrogen in the rats was significantly reduced after inhibiting TRPM7. (*p* < .05) (Figure 5D).

### 4.8 Effects of sevoflurane on apoptosis of renal tissue cells after regulating the expression of TRPM7 protein

**Figure 6:**
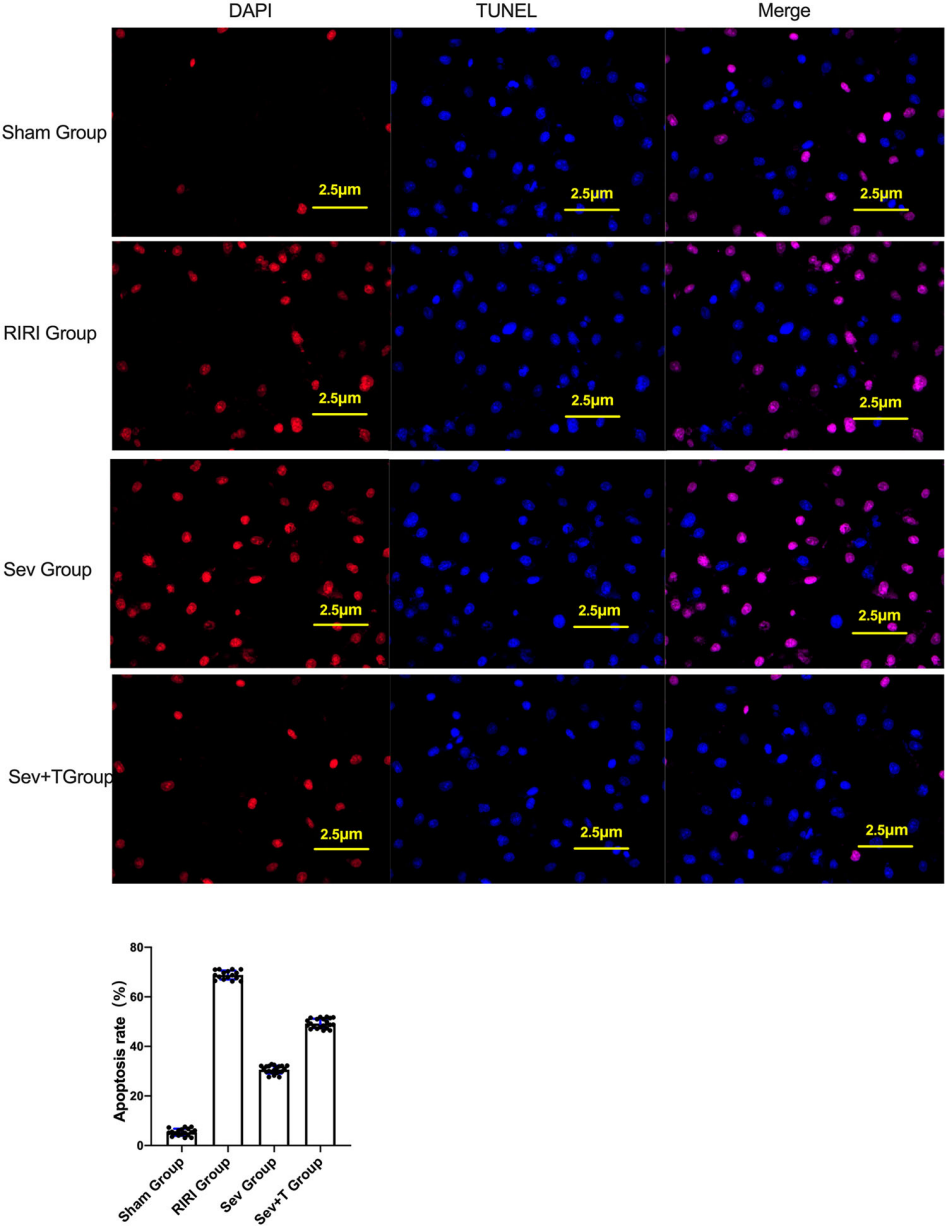
Effect of sevoflurane on apoptosis of renal tissue after regulating TRPM7 protein expression. (A) TUNEL apoptosis assay. (B) Comparison of apoptosis rate of rats in each group. RIRI group: renal ischemia‐reperfusion injury group; Sev group: sevoflurane treatment group; Sev + T group: sevoflurane treatment group after inhibiting the expression of TRPM7; Sham group: sham operation group. *p < .05 compared to the Sham group. ^#^ *p* < .05 compared with RIRI group. ^&^ *p* < .05 compared with Sev group.

**Figure 7:**
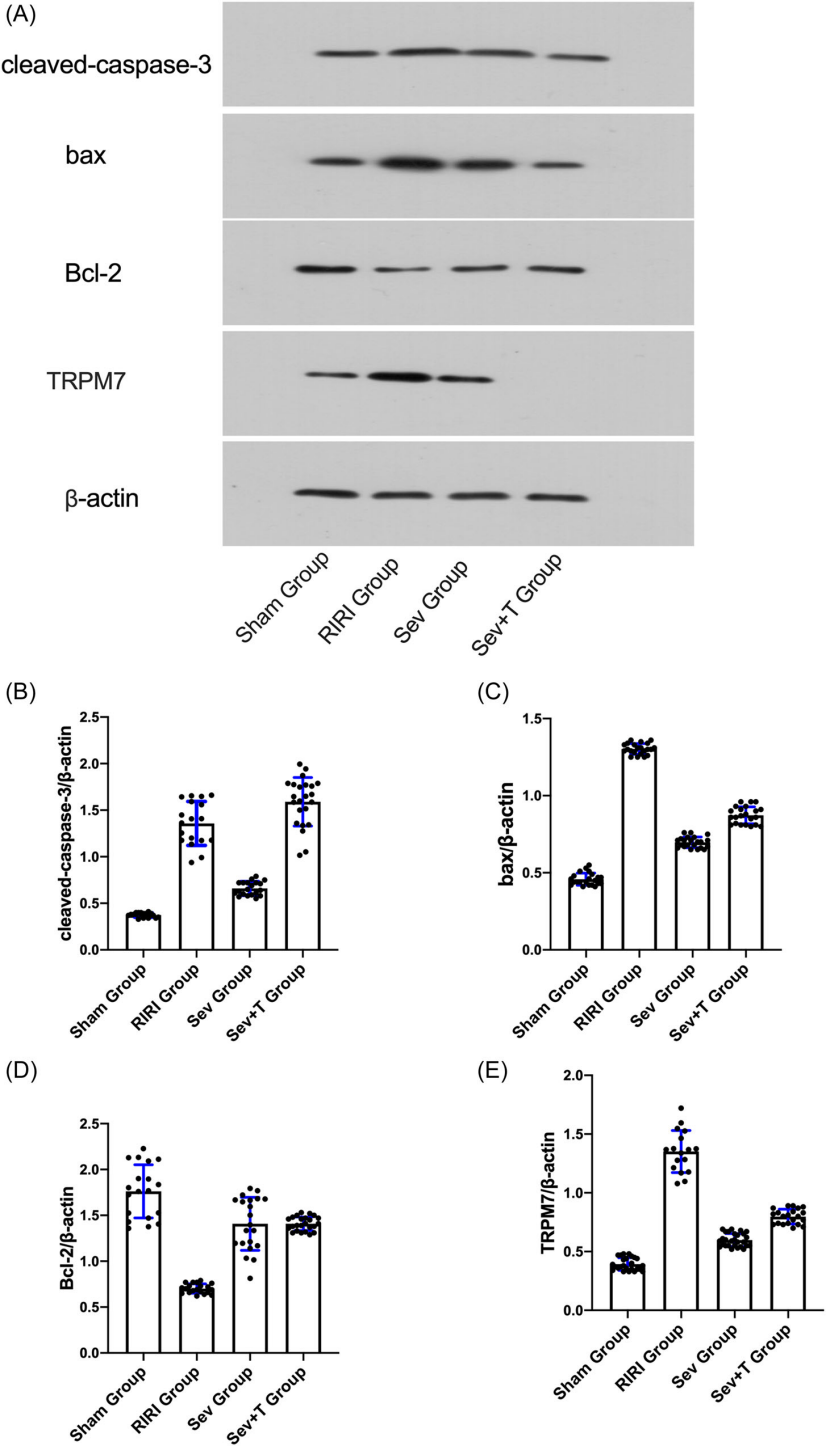
Apoptosis‐related protein and TRPM7 protein expression. (A) Western blot; (B) Expression of apoptosis‐related protein cleaved‐caspase‐3; (C) Expression of apoptosis‐related protein bax. (D) Expression of apoptosis‐related protein Bcl‐2. (E) TRPM7 protein expression. RIRI group, renal ischemia‐reperfusion injury group; Sev group, sevoflurane treatment group; Sev + T group, sevoflurane treatment group after inhibiting the expression of TRPM7; Sham group, sham operation group. *p < .05 compared to the Sham group. ^#^ *p* < .05 compared with RIRI group. ^&^ *p* < .05 compared with Sev group.

The TUNEL cell apoptosis test showed that compared with the Sham group, the apoptosis rate of renal tissue in the RIRI group, the Sev group and the Sev + T group was increased (*p* < .05). Compared with the RIRI group, the apoptosis rate of renal tissue in the Sev group was lower than that in the RIRI group (*p* < .05). After inhibiting TRPM7, the effect of sevoflurane on reducing the apoptosis of the rats was significantly reduced (*p* < .05) (Figure 6A,B). WB results showed that compared with the Sham group, the expressions of apoptosis‐related proteins cleaved‐caspase‐3 and bax in the kidney tissue of the RIRI group, Sev group, and Sev + T group were increased (*p* < .05), and the expression of Bcl‐2 was decreased (*p* < .05). *p* < .05). Compared with the RIRI group, the expressions of apoptosis‐related proteins cleaved‐caspase‐3 and bax in the kidney tissue of the Sev group were lower than those of the RIRI group (*p* < .05), and the expression of Bcl‐2 was higher than that of the RIRI group (*p* < .05). The apoptosis of kidney tissue of rats in RIRI group and Sev group increased. After inhibiting TRPM7, the effect of sevoflurane on reducing apoptosis in rats was significantly reduced (*p* < .05) (Figure 7A–E). Sevoflurane can ameliorate the apoptosis of renal cells induced by renal ischemia‐reperfusion, and this renoprotective effect of sevoflurane is significantly attenuated after inhibition of TRPM7.

### 4.9 The effect of sevoflurane on serum oxidative stress‐related indicators and serum inflammatory factor levels

**Figure 8:**
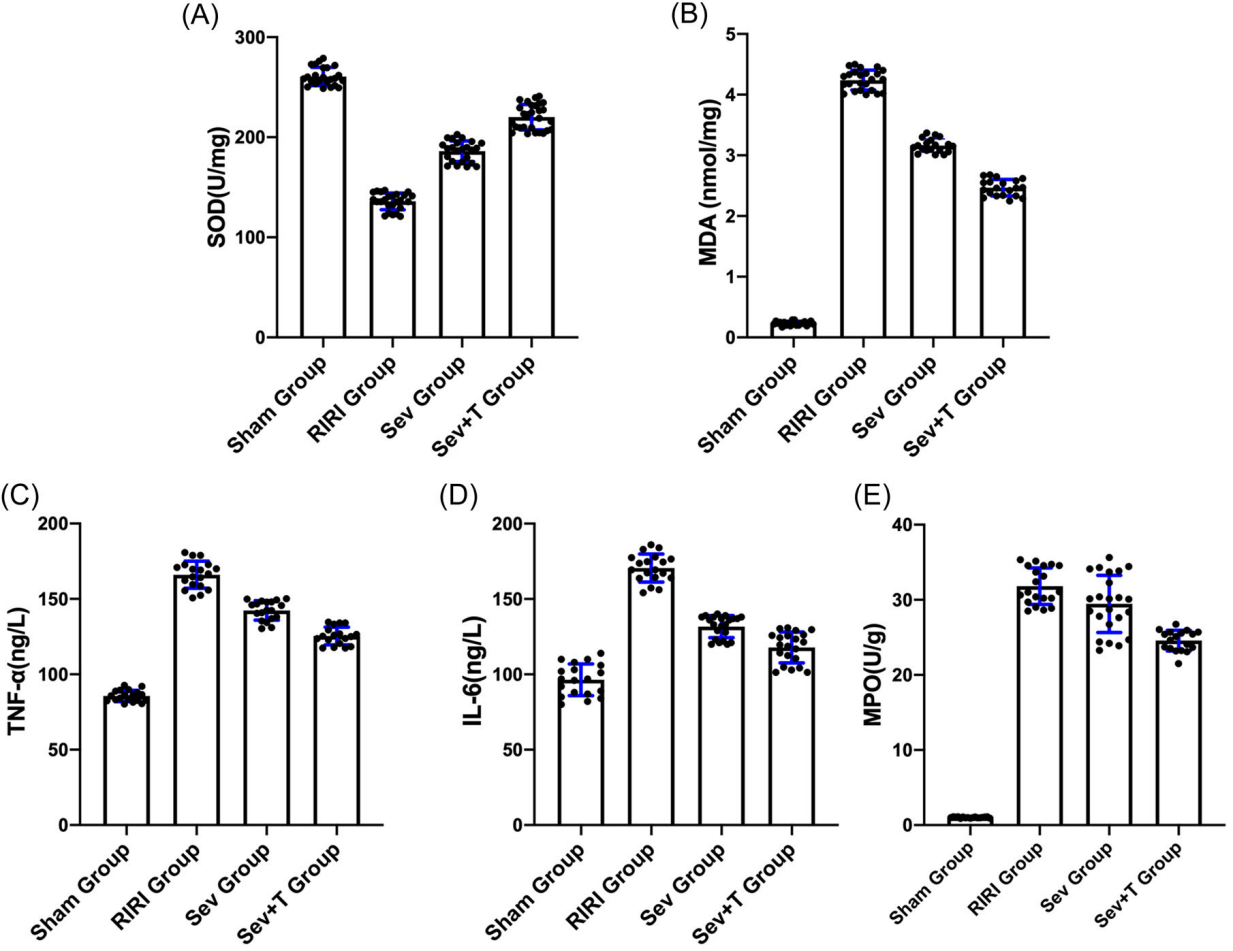
The effect of sevoflurane on serum oxidative stress‐related indicators and serum inflammatory factor levels. (A) Changes in SOD, an oxidative stress‐related index. (B) Changes of oxidative stress‐related index MDA. (C) Changes in inflammatory factor IL‐6. (D) Inflammation changes due to TNF‐α. (E) Changes in inflammatory factor MPO. IL‐6, interleukin‐6; MDA, malonaldehyde; MPO, myeloperoxidase; RIRI group, renal ischemia‐reperfusion injury group; Sev group, sevoflurane treatment group; Sev + T group, sevoflurane treatment group after inhibiting the expression of TRPM7; SOD, superoxide dismutase; Sham group, sham operation group; TNF‐α, tumor necrosis factor‐α. *p < .05 compared to the Sham group. ^#^ *p* < .05 compared with RIRI group. ^&^ *p* < .05 compared with Sev group.

Compared with Sham group, the expressions of oxidative stress‐related indexes SOD and MDA in serum of rats in RIRI group, Sev group and Sev + T group were increased (*p* < .05). The expressions of oxidative stress‐related indexes SOD and MDA in the serum of the Sev group were lower than those of the RIRI group (*p* < .05). After inhibiting TRPM7, the effect of sevoflurane on reducing the serum oxidative stress level of the rats was significantly reduced (*p* < .05). Compared with the Sham group, the expressions of inflammatory factors IL‐6, TNF‐α and MPO in the serum of the RIRI group, Sev group and Sev + T group were increased (*p* < .05). The expressions of oxidative stress‐related indicators IL‐6, TNF‐α, and MPO in the serum of the rats in the Sev group were lower than those in the RIRI group (*p* < .05). After inhibiting TRPM7, the effect of sevoflurane on reducing the levels of serum inflammatory factors in rats Significantly decreased (*p* < .05) (Figure 8A–E). In conclusion, sevoflurane can improve serum oxidative stress and inflammatory response induced by renal ischemia‐reperfusion, and this protective effect of sevoflurane is significantly attenuated after inhibition of TRPM7.

## 5 DISCUSSION

Compared with other inhaled anesthetics, sevoflurane has the advantages of rapid induction, less respiratory irritation, low solubility, better hemodynamic stability, rapid absorption and clearance, and rapid and complete recovery. The advantage is that it is a relatively safe and ideal inhalation anesthetic.^21^, ^22^

In recent years, studies have found that sevoflurane can reduce the ischemia‐reperfusion injury of the heart, brain, lung, liver, and other organs. In this study, an acute renal ischemia‐reperfusion injury model was constructed through animal experiments, renal function was detected, and pathological changes in renal tissue were observed to explore the protective effect and possible mechanism of sevoflurane on renal ischemia‐reperfusion injury. The experimental results show that: sevoflurane regulates the expression of TRPM7 protein, controls the process of apoptosis, further significantly reduces serum creatinine and blood urea nitrogen levels, and increases SOD activity, which can play a role in resisting acute renal ischemia‐reperfusion injury. At present, there is still no effective method to prevent and treat renal ischemia‐reperfusion injury in clinical practice. Therefore, it is an urgent clinical need to deeply explore the pathogenesis of sevoflurane and RIRI, so as to find an effective method to prevent and treat the degree of RIRI. This study was modeled according to the previous literature on ischemia reperfusion for the study of this experiment.^20^

Inflammatory response is a key factor in the development of RIRI.^23^ Renal RIRI is often accompanied by the accumulation of a large number of neutrophils, and the inflammatory factors, chemokines and adhesion molecules produced by the inflammatory response can further activate neutrophils, and chemotaxis make them infiltrate and aggregate.^24^, ^25^ Inflammatory factors such as TRPM7 can activate immune cells, promote the secretion of IL‐6, and so forth, initiate a cascade reaction, promote the adhesion of vascular endothelial cells and neutrophils, lead to obstruction of vascular permeability, aggravate the inflammatory response, and further aggravate renal injury. Therefore, the expression of TRPM7 indicates the intensity of the inflammatory response in renal tissue.^26^, ^27^, ^28^ Some scholars' studies have found that sevoflurane pretreatment can improve myocardial tissue damage and reduce the levels of TNF‐α and IL‐6.^29^ The results of this study show that sevoflurane pretreatment can reduce the inflammatory response during renal RIRI, inhibit oxidative stress and reduce renal tissue damage, and sevoflurane combined with TRPM7 inhibitor treatment can further reduce the inflammatory response during renal RIRI.

The inflammatory response regulation mechanism of renal RIRI process is relatively complex, and TRPM7, as an inflammatory switch, can activate the body's immune inflammatory response by regulating the RP‐1/NAD + pathway to regulate the necroptosis signaling pathway.^26^, ^30^ Necroptosis, as an important signaling factor in TRPM7, binds to the inhibitory protein as an inactive tetramer under normal physiological conditions, but under the action of IRI, the inhibitory protein is degraded, and after necroptosis is separated, it is transferred to the nucleus alone, inducing the body. A large number of inflammatory factors are released, triggering a waterfall inflammatory response.^31^ The results of this study also found that sevoflurane preconditioning combined with TRPM7 inhibitor can down‐regulate the expression of TNF‐α and IL‐6, and reduce the level of oxidative stress, suggesting that sevoflurane preconditioning can inhibit the activation of TRPM7 signaling pathway, thereby reducing renal IRI. The results of this study are consistent with the above‐mentioned findings.

In addition, this study has many shortcomings. First of all, relevant in vitro experiments have not been carried out for further verification. Second, the specific molecular mechanism that sevoflurane regulates the expression of TRPM7 and participates in the protection of rat renal ischemia‐reperfusion injury has not yet been explored. These will be further improved in subsequent experiments.

In summary, through this experimental study, we found that sevoflurane participates in the protection of rat renal ischemia‐reperfusion injury by downregulating the expression of TRPM7, which provides a basis for further clinical trials of sevoflurane in renal ischemia‐reperfusion injury protection new direction.

## AUTHOR CONTRIBUTIONS

Xudong Xu and Rongrong Deng conceived the study and designed the experiments. Rongrong Deng, Lu Zou, and Xiaoyan Pan contributed to the data collection, Zhifeng Sheng, Da Xu, and Tingting Gan performed the data analysis and interpreted the results. Xudong Xu wrote the manuscript; Xudong Xu and Rongrong Deng contributed to the critical revision of article. All authors read and approved the final manuscript.

## CONFLICT OF INTEREST

The authors declare no conflict of interest.

## Data Availability

The data that support the findings of this study are available from the corresponding author (Xudong Xu), upon reasonable request.
